# An Acute Model of Care to Guide Eating & Drinking Decisions in the Frail Elderly with Dementia and Dysphagia

**DOI:** 10.3390/geriatrics3040065

**Published:** 2018-10-02

**Authors:** Dharinee Hansjee

**Affiliations:** Lewisham & Greenwich NHS Trust, Queen Elizabeth Hospital, London, SE18 4QH, UK; dharinee.hansjee@nhs.net

**Keywords:** aspiration, dysphagia, risk feeding, dementia

## Abstract

People with dementia frequently develop dysphagia (swallowing impairment), which causes them to be at high risk of aspiration pneumonia, resulting in hospital admissions. These individuals are advised against alternative nutrition and hydration as this does not eliminate the risk of developing chest infections. The purpose of this study was to establish the impact on length of stay by having a protocol to guide eating and drinking despite aspiration risks (risk feeding). A risk-feeding protocol was developed and implemented in a hospital setting. The quality improvement methodology of Plan-Do-Study-Act (PDSA) was employed to evaluate the impact of a protocol on the length of stay in patients with dementia and aspiration pneumonia. Annual audits (2016–2018) on the time taken from admission to when a route of nutrition was established were conducted, with adaptations made to the protocol. There was a reduction in nutrition planning times with each year. On closer evaluation of the data, improved nutrition planning times for this cohort impacted on a shorter length of stay. Having a model of care in place to guide feeding decisions in dementia coordinates care, as demonstrated in timely decision-making. For patients who are admitted with aspiration pneumonia and dementia, a decreased length of stay is evident.

## 1. Introduction

The National Health Service (NHS) spends around £820 million a year treating older patients who no longer need to be in hospital, with the cost of care per older person per day outlined to be £303 [1]. The prevalence of dysphagia (swallowing impairment) in the elderly can reach up to 93%, increasing with the degree of frailty and dependence [2]. For individuals greater than 75 years, the risk of pneumonia (chest infections) due to dysphagia, is six times greater than those 65 years of age [2]. Cabre et al. [3] found that pneumonia was the principal cause of acute hospital admission in 55% of people living with dementia.

The familiar model of care for people with advanced dementia and dysphagia is the revolving door of recurrent chest infections, frequently associated with aspiration and related readmissions. There are individuals with dementia who resist or are indifferent to food, fail to manage the food bolus adequately once it is in the mouth (oral phase dysphagia) or aspirate when swallowing (pharyngeal phase dysphagia). There are also many contributory factors such as poor oral hygiene, high dependency levels for being positioned and fed as well as the need for oral suctioning [4,5].

The challenge arises when these individuals are deemed unsuitable for alternative nutrition or hydration (ANH) following a multidisciplinary team (MDT) discussion, usually involving the medical team, the dietician and the speech and language therapist. A patient may be inappropriate for ANH if the procedure risk outweighs the benefit; the patient themselves decline ANH or there is poor prognosis/a short life expectancy as evident in advanced dementia [6]. Clinicians are then faced with the dilemma of how best to manage these patients who are unsuitable for ANH but at risk of choking on food/fluid and developing an aspiration pneumonia.

While tube feeding might therefore be considered a safer option, a Cochrane Review [7], revealed insufficient evidence to suggest tube feeding is beneficial in people with advanced dementia; and the National Institute for Health & Care Excellence (NICE) [8] recommend tube feeding in patients with advanced dementia only where dysphagia is thought to be transient in an acute illness.

In current practice, the preferred option therefore, for individuals with advanced dementia, is to continue eating and drinking orally despite the risk of developing chest infections [9,10]. This choice can be referred to as risk feeding or eating and drinking at risk. Despite the clarity in the literature, as outlined above, the decision-making process regarding whether to introduce tube feeding or to eat and drink at risk continues to remains a challenge for professionals as well as the individuals concerned and/or their carers [11].

According to Puntil-Sheltman [12], the clinical reasons are based on whether the intervention is beneficial or burdensome to the patient while the moral arguments are usually based on the person’s perceived quality of life. According to the NICE [8], the person’s individual beliefs, preferences, needs and best interests should be central to the decision-making process.

Decisions on nutritional options as a person approaches the end of life are ethically complex, particularly if the individual lacks decision-making capacity [11]. The lack of guidance around decision-making can compromise quality and safety of care, resulting in poor patient outcomes and increased length of in patient stay [13].

An initial retrospective audit conducted locally in March 2011, first highlighted the gaps in the clinical decision-making process. The audit was based on seven patients admitted with aspiration pneumonia who were referred to speech and language therapy (SLT) over the month. The audit was carried out on an elderly care ward (patients over 65 years of age), in an Acute NHS Trust. All seven patients did not have a formal diagnosis of dementia but were reported to be confused. Frailty was screened on admission, using the Rockwood Clinical Frailty Scale [14], validated as a predictor of outcomes for older people. All patients had a clinical frailty score of above 6 which indicated a moderate to severe degree of dependence in meeting their activities of daily living. Cognition was not formally assessed. There was no routine screening of swallowing in place at this Trust. Although these individuals presented with high risk factors for developing aspiration pneumonia, not all were referred to SLT on admission.

Medical case notes were used to establish the number of days taken, from admission to the hospital, to when a nutrition plan was put in place. Nutrition plan refers to the decision to eat and drink orally, continue oral intake despite risks of aspiration or ANH. Pre-admission diet and fluid information was not retrieved.

The crucial finding from the medical entries suggested delays of more than one day (2–14) in nutrition planning for five out of the seven patients. There was a mean value of 6 days before a nutrition plan was put in place. Analysis of the bedside swallow assessment findings in the medical notes, revealed these patients to demonstrate clinical signs of aspiration on all consistencies/textures trialed and therefore at high risk for developing of aspiration pneumonia. An MDT discussion was essential to establish a plan for nutrition. The time taken towards clinical decision-making resulted in significant delays of which the primary source of delay was the assessment of capacity.

The other key finding related to the inconsistencies of the diet regime for this cohort population. Some patients were placed on a regular diet and thin fluid and referred to SLT when there was reduced oral intake while others were left nil by mouth with intravenous fluids, compromising safety and comfort. These findings highlighted the need to introduce a process to better manage nutrition and hydration in this patient group. The results of the small-scale audit led to the inception of a protocol to guide timely decision-making for individuals eating and drinking at risk in the acute setting.

The purpose of this study is to outline the steps on the development and implementation of the protocol to guide eating and drinking at risk. An evaluation of the impact of the protocol on length of stay in an acute hospital setting will further be discussed.

## 2. Methods

### 2.1. Development of a Protocol

The parameters for the risk-feeding protocol were established from the gaps in practice disclosed by the audit. The document incorporates the reasons why a person may be a candidate for risk feeding. To determine if the individual has the capacity to decide regarding the route of their nutrition, their mental capacity is assessed using the four domains within the Mental Capacity Act (2005) [15]. If the individual does not have capacity, discussions will need to occur with the next of kin/spouse/family member. If there is no next of kin or advance care plan in place, an independent mental health advocate will be consulted. These are options included on the document. There is the authorization endorsed by the signatures of the consultant and the speech and language therapist prompting MDT discussions information sharing with the patient/family. Risk reducing diet recommendations are also included to ensure the patient receives the appropriate diet and fluids. This would be completed following a formal clinical bedside assessment of the swallow.

Once this document was designed, it was disseminated to the hospital Trusts consultants, governance boards and ethics committee for consultation. This was not a research ethics committee but rather a special interest group for discussions on cases and learning in the field of medical ethics. The layout and wording of the document was reviewed by a palliative care consultant. The document was ratified by the Trust’s legal department and Quality and Safety forum.

### 2.2. Implementation and Change Management

Key stakeholder engagement was the first step in the development of a protocol to guide feeding decisions. A palliative care consultant assisted in the layout and wording of this document. The guidelines were drafted and disseminated to the Trust’s ethics committee and legal department and finally to the acute medicine, long term conditions and surgery governance forums.

According to Buchanan and Dawson [16], the best solutions to problems when working in a complex system come from those who are constantly communicating with one another at ground level. Steps were therefore taken to ensure spread of the innovation. The risk-feeding protocol was presented at the Corporate Nutrition Steering Group. Talks were held at academic half days and grand rounds for medical staff. Training sessions were scheduled for junior doctors on the admitting ward. Bite size (15 min) training sessions were held at the nurses’ stations on the ward to prevent nurses/health care assistants having to be released to attend training. Presentations were delivered at nutrition events across the Trust. The speech and language therapists were invited onto rolling educational programs for both the doctors on and nurses on the elderly care wards.

### 2.3. Audit/PDSA (Plan-Do-Study-Act)

PDSA cycle is a quality improvement structure used to test out small-scale changes on a system and then review it before deciding how to proceed. The principle of the PDSA is not about having the perfect solution but about trying things out and amending it accordingly [17]. The approach seemed applicable to this study as the method is noted to effect change in small-scale quality improvement projects. Remedial action via PDSA audit cycles were conducted with additions and amendments made to the protocol.

For quality and audit purposes, patients who were risk feeding, having undergone a clinical beside assessment of swallow and the shared decision-making process of the protocol, are placed on a database. Their name, primary diagnosis, the reason for risk feeding, diet/fluid recommendations and re-admission dates are recorded. Information from the database and the electronic patient records was used to audit the time taken from admission to when a nutrition plan was put in place for individuals deemed suitable for risk feeding.

The audit was conducted retrospectively over a month each year (2016–2018). The data was reviewed from 2016, when a six-day SLT service was in operation. Only individuals with a diagnosis of dementia, admitted with aspiration pneumonia were included in the audit. All patients included in the audit had a clinical frailty score of above 6 indicating a moderate to severe degree of dependence. The numbers recruited over the month of April each year were: 7 in 2016, 8 in 2017 and 6 in 2018, including a total of 21 patients. Nutrition planning times from admission to the hospital to when a nutrition plan was put in place were extrapolated from the electronic patient records (EPR). Length of stay calculations were also retrieved, for this cohort, from the EPR system for patients who were risk feeding with a diagnosis of dementia. The EPR system was in operation from 2014, therefore there is no length of stay data recorded for the initial audit conducted in 2011.

## 3. Results

Prior to a protocol being implemented, from the audit conducted in 2011, there were average delays of six days (ranging from 0–14) in putting a plan of nutrition in place (see Table 1). Subsequent annual audits following the introduction of this protocol resulted in improved nutrition planning times.

**Figure 1:**
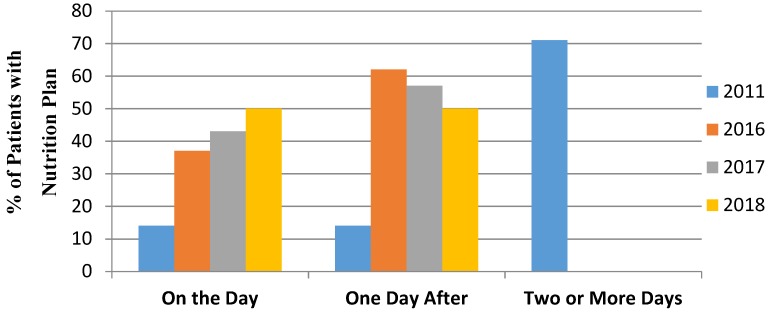
Nutrition Planning Times from Admission.

A six-day SLT service commenced in 2016. Figure 1 reveals 37% of individuals having had a plan in place on the day of admission in 2016, which increased to 43% in 2017 and 50% in 2018. From the data reviewed, 100% of individuals had a nutrition plan in place within a day after admission.

For both audits conducted over 2017 and 2018, 15% of individuals avoided admission. These patients were assessed in the Accident and Emergency Department, had a plan of nutrition set up and were discharged back to the community with a PEACE (Proactive Elderly Advance CarE) plan [18]. The PEACE plan refers to a document outlining the person’s preferences or in some cases the best interest decisions and future planning as agreed by a General Practitioner (GP) and care/nursing homes [19].

As reflected in Table 2 below, quality of life was the primary reason for eating and drinking at risk for 71% of these individuals. The international dysphagia descriptors were used to describe diet and fluid selections. There were 80% who were placed on a puree diet and 66% on thin fluids.

**Figure 2:**
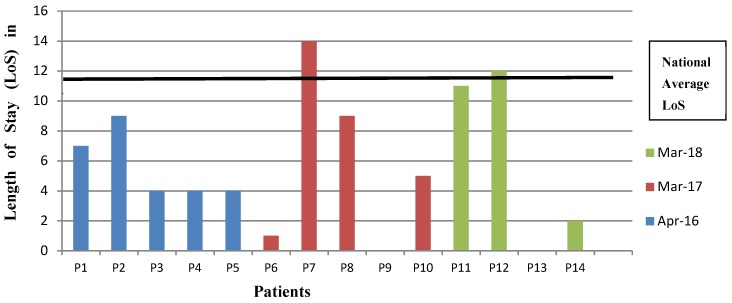
Length of Stay against the National Average.

From the 21 patients selected from a month over the respective years (2016–2018), there were 14 who were admitted with aspiration pneumonia and a diagnosis of dementia. Figure 2 indicates the average length of stay for these individuals to have been 6 days (ranging from 0–14).

## 4. Discussion

A risk-feeding protocol was devised to guide acute teams through an organized decision-making process, encompassing patient choice and MDT input. A considerable learning point in the process of implementation was the need to have a risk-feeding policy in place to accompany the roll-out of the protocol. According to Dixon-Woods et al. [20], one needs to be explicit about the innovation and what mechanisms are at work to avoid the “cargo cult quality improvement” where initiatives are implemented without proper understanding. The complexity of implementing a risk-feeding pathway lies in engagement and ownership of the individual roles of the MDT to ensure the pathway is robust. According to Bate et al. [21], the key to quality is found in the processes that connect them. Although errors or failings should be picked up and improved upon using robust systems improvement, it is also imperative to create a learning culture to ensure sustenance of improved systems within this ongoing, emergent process. The policy was therefore crucial in outlining a structure for dissemination and training. The document was beneficial in empowering the hospital team and wider pathways in understanding the purpose, scope, and their role within the risk-feeding process.

Through the series of PDSA audit cycles there were developments made to the protocol and model of care. Using the risk-feeding protocol, open discussions are had about the risks associated with eating and drinking, which forms an essential part of the individual’s care [22]. According to NICE guidelines QS1 [8], decision-making around feeding should take into account individual preferences. A protocol to guide feeding decisions, will allow people with dementia and their carers, a choice on decisions affecting care.

In addition, information leaflets explaining the management options and risks were devised to support the individual or their carers in making informed decisions about their nutrition. The protocol stimulates a problem-solving approach from the MDT and individual/significant other. There are discussions with the nurses, doctors, and dietitians but also involvement from physiotherapy regarding chest management and establishing a ceiling of care. The palliative care team provides input on end-of-life care, while social services and discharge teams are proactively involved in the consideration of risk feeding within discharge planning.

As this is a population group at high risk of aspirating, a review of their medication is essential [23]. A prompt for a medication review was therefore added to the original risk-feeding protocol to ensure medication is provided in a form that is easier to swallow. In this way, risks of possible aspiration of medication are also considered. This review could be carried out by a pharmacist in the acute setting or a GP, on discharge.

One further addition to the protocol through PDSA audit cycles was the consideration of an advance care plan, if appropriate. The risk-feeding process invites the MDT and individual to discuss future management in the form of an advance care plan or PEACE document. For individuals who experience recurrent aspiration pneumonia-related admissions, this has led to clarity of personal/family wishes and empowers the individual and/or their carers to be involved in their care, while simultaneously allowing the professional to improve end-of-life care for that individual [24]. The documentation of decisions on current and future nutritional management allows accurate handover to receiving teams, such as GPs, care homes, and health and social care services [25].

It is evident that through the amendments and adaptations to the protocol there is now a nutrition plan in place for 100% of patients who are candidates for eating and drinking at risk, a day following admission. For patients with a diagnosis of dementia, admitted with aspiration pneumonia, the protocol enhances timely and coordinated discussions resulting in a decreased length of stay. This model of care can be added to the nutrition component of the comprehensive geriatric assessment contributing to the holistic management of the frail elderly.

Hospital admission and increased length of stay often has a negative effect on the health and wellbeing of people with dementia [26]. The average length of stay for a person with dementia in an acute setting is 11.8 days [26]. Patients who are kept nil by mouth for their whole hospital stay still have a very high pneumonia and mortality rate [27]. Having a protocol in place, averages the length of stay in hospital to six days, while supporting a person-centered decision. It is a challenge to establish if it was the MDT working or the protocol which reduced length of stay as the protocol enhances and formalizes team discussions. Guidance on eating and drinking at risk has the potential to improve fiscal outcomes and the quality of life of the individual.

What is needed however to prevent the emergency admissions in this cohort, is for these discussions to occur in the individuals own home care/nursing home with a documented plan to prevent further chest related readmissions [25].

## 5. Limitations of the Study

The primary limitation of this study was the small sample sizes through all the audits conducted over the respective years. Although there were low numbers in the initial audit, the audit did raise significant findings which the protocol has been evidenced to address.

There was no length of stay audit data for the 2011 review to compare with the audits conducted between the years 2016–2018.

Although the data in the study was based on the dementia population, the protocol has been found to be beneficial in several patient groups and for individuals either approaching the end of their life or choosing to eat and drink with consent. 

## 6. Implications for Further Research

Research involving a consultation with multi-professional experts on a protocol for the community setting will be essential in meeting the vision of moving care closer to home.

## 7. Conclusions

The protocol promotes robust communication between the acute and community settings which is essential for a safer and coordinated discharge [18]. Having a protocol in place reduces prolonged admissions in the frail elderly, leading to a better quality of life for these individuals, with accruing cost savings to the health service. The innovation of the risk-feeding protocol is manifested in the use of existing resources and services towards an organized, improved way of working making this model of care easily transferable to healthcare settings nationally and globally.

## Figures and Tables

**Table 1:** Nutrition Planning Times.

Patients	Number of days from admission to nutrition plan
P1	14
P2	11
P3	10
P4	7
P5	2
P6	1
P7	0

**Table 2:** Diet /Fluid Recommendations and Reasons for Risk Feeding.

Patient	Diet (L = Level)	Fluid (L = Level)	Reason for Risk Feeding
P1	Puree (L4)	Thin (L0)	Quality of life
P2	Puree (L4)	Thin (L0)	Quality of life
P3	Puree (L4)	Thin (L0)	Palliative Care
P4	Puree (L4)	Mildly Thick (L2)	Palliative Care
P5	Puree (L4)	Mildly Thick (L2)	Quality of life
P6	Puree (L4)	Slightly Thick (L1)	Quality of life
P7	Regular (L7)	Thin (L0)	Quality of life
P8	Puree (L4)	Thin (L0)	Quality of life
P9	Puree (L4)	Thin (L0)	Quality of life
P10	Puree (L4)	Thin (L0)	Procedure risk (ANH)outweighs benefit
P11	Puree (L4)	Thin (L0)	Patient declined ANH
P12	Regular (L7)	Thin (L0)	Patient declined modified diet and fluids
P13	Puree (L4)	Thin (L0)	Patient refused thickened fluids
P14	Puree (L4)	Thin (L0)	Quality of life
P15	Minced and moist (L5)	Mildly Thick (L2)	Procedure risk (ANH) outweighs benefit
P16	Regular (L7)	Thin (L0)	Quality of life
P17	Puree (L4)	Thin (L0)	Quality of life
P18	Puree (L4)	Mildly Thick (L2)	Quality of life
P19	Puree (L4)	Thin (L0)	Procedure risk outweighs the benefit
P20	Puree (L4)	Slightly thick (L1)	Quality of life
P21	Puree (L4)	Thin (L0)	Quality of life
